# Solitary Porokeratoma on the Foot: A Case Report and Literature Review

**DOI:** 10.7759/cureus.105074

**Published:** 2026-03-11

**Authors:** Igor Shendrik, Chance Morris, Neil Crowson, Svetlana Bobkova

**Affiliations:** 1 Dermatopathology, Pathology Laboratory Associates, Tulsa, USA; 2 Dermatology, INTEGRIS Health Medical Group, Oklahoma City, USA; 3 Biomedical Sciences, Oklahoma State University Center for Health Sciences, Tulsa, USA

**Keywords:** dyskeratoma, porokeratoma, porokeratosis, porokeratosis ptychotropica, verrucous carcinoma

## Abstract

Porokeratoma (PRKT) is a rare epidermal acanthoma that can clinically resemble other hyperkeratotic or verrucous lesions, creating potential diagnostic uncertainty without histopathologic evaluation. It typically presents as a solitary exophytic lesion that lacks the annular architecture associated with other porokeratosis variants and may therefore be misclassified on clinical examination alone. We report the case of an immunocompetent 67-year-old male with a 10-week history of a progressively enlarging hyperkeratotic nodule on the dorsal aspect of the right ankle. His examination revealed a well-demarcated lesion without annular features or surrounding satellite lesions. Histopathologic analysis following saucerization demonstrated acanthosis, papillomatosis, compact orthokeratosis, and multiple confluent cornoid lamellae with associated hypogranulosis and dyskeratotic keratinocytes, confirming the diagnosis of PRKT. This case underscores the importance of histopathologic assessment in distinguishing PRKT from other clinically similar verrucous lesions to support accurate diagnosis and guide appropriate management.

## Introduction

Porokeratoma (PRKT) is a rare epidermal neoplasm defined by multiple confluent cornoid lamellae distributed throughout a hyperkeratotic lesion. Histologically, these cornoid lamellae are composed of columns of parakeratosis overlying dyskeratotic or vacuolated keratinocytes with loss of the underlying granular layer. First described by Walsh et al. in 2007, PRKT was proposed as a distinct acanthomatous lesion with porokeratosis (PK)-like cornoid lamellation but without the annular architecture of classic PK [1]. Unlike conventional PK, PRKT lacks central epidermal atrophy and does not show cornoid lamellae restricted to the lesion periphery. Additional common features include acanthosis, papillomatosis, and compact orthokeratosis, with occasional dyskeratotic keratinocytes and chronic lymphohistiocytic infiltrate. Reported cases have predominantly involved middle-aged or older men, with lesions most often presenting on the extremities as solitary papules, plaques, or nodules. Although several other PK variants have been described (including PK of Mibelli, disseminated superficial actinic PK, PK plantaris, palmaris et disseminata, linear PK, and PK ptychotropica), these entities differ clinically and histologically despite their shared feature of cornoid lamellation [2]. Clinically, PRKT may resemble a viral wart, seborrheic keratosis, warty dyskeratoma, inverted follicular keratosis, verrucous carcinoma, or hypertrophic lichen planus. Histopathologically, it may also mimic old verruca vulgaris or PK ptychotropica. However, PK ptychotropica usually presents as symmetric hyperkeratotic plaques in the gluteal or perianal region with a characteristic butterfly-shaped distribution, whereas verruca vulgaris shows viral cytopathic change and hypergranulosis. Warty dyskeratoma and inverted follicular keratosis are distinguished by cup-shaped epidermal invaginations, acantholytic dyskeratosis, and peripheral basaloid proliferation, features absent in PRKT. The etiology of PRKT remains uncertain; although associations with ankylosing spondylitis, chronic lymphocytic leukemia, and human papillomavirus (HPV) infection have been reported, these may be incidental given the small number of cases described to date [3].

## Case presentation

**Figure 1:**
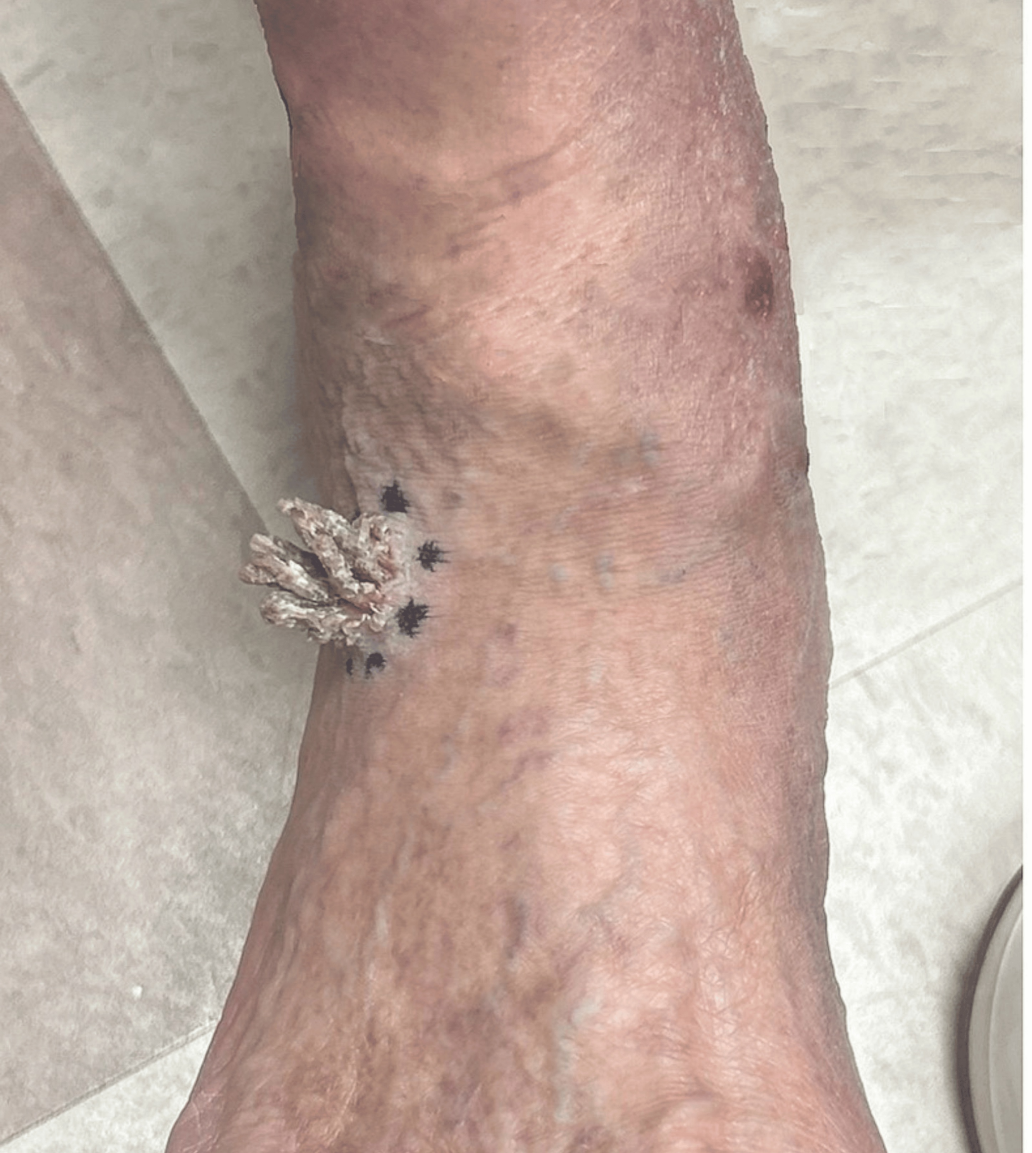
Solitary, exophytic keratotic papule on the ankle with clustered horn-like projections and dry adherent scale. The surrounding skin shows atrophy and varicosities. The dotted marks indicate the biopsy edges.

A 67-year-old male presented to our dermatology clinic with a 10-week history of a solitary growth on the right foot. The lesion had slowly enlarged and occasionally bled when traumatized, but was otherwise asymptomatic. There was no personal or family history of PK, verrucous epidermal lesions, or immunosuppressive disorders. There was no history of trauma or chronic sun exposure. On examination, a solitary, well-demarcated, firm, exophytic keratotic papule was identified on the ankle near the malleolar region. The surface demonstrated a tightly aggregated array of vertically oriented keratinaceous columns, imparting a spiculated, horn-like appearance without the formation of a single confluent cutaneous horn. The surrounding skin exhibited background xerosis with associated atrophy and varicosities (Figure 1).

The surface of the lesion was verrucous with overlying hyperkeratosis. No satellite lesions, annular plaques, or peripheral ridges were identified. Regional lymph nodes were not enlarged. The remainder of the cutaneous examination was unremarkable. The lesion was excised under local anesthesia and measured 2.1 cm in greatest dimension; it was well demarcated. The specimen was submitted for routine histopathologic evaluation.

Microscopic examination of the biopsy sample demonstrated a papillomatous epidermal tumor with tall digitations projecting above the surface. The lesion was centered in the epidermis without destruction of the dermis. Numerous narrow parakeratotic columns manifested as vertical streaks from the surface into the keratin layer. Below parakeratotic columns, dyskeratosis, and loss of the granular layer were evident, features diagnostic of cornoid lamellae. There was no central epidermal atrophy. Mild vascular dilatation with a surrounding lymphoid infiltrate is seen within the superficial dermis. No koilocytosis typical of HPV infection was seen. No significant cytologic atypia or mitoses were identified in the examined fields.

There is a papillated epidermal tumor with broad acanthosis and numerous vertically oriented parakeratotic columns consistent with multiple cornoid lamellae distributed throughout the lesion. The process remains confined to the epidermis with a mild superficial lymphocytic infiltrate.

**Figure 2:**
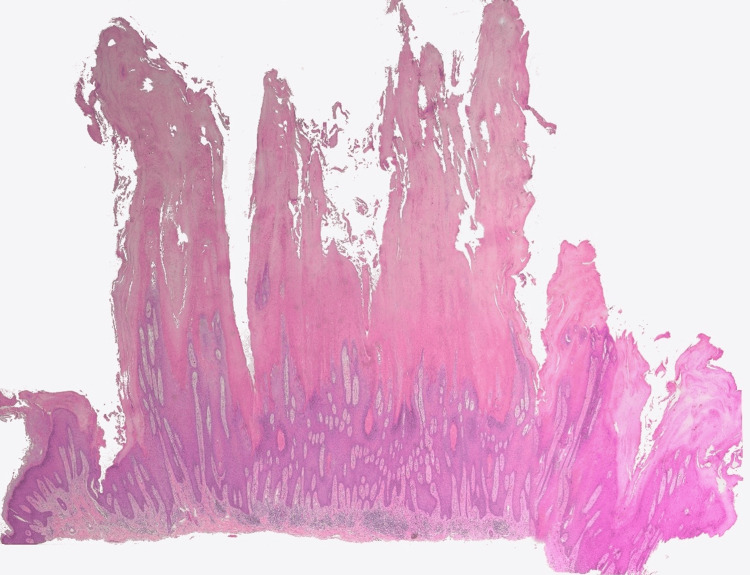
Panoramic H&E section assembled from 20 partially overlapping fields (5 × 4 grid) captured at 2× objective and stitched into a single mosaic. A uniform, global brightness/white-balance normalization was applied.

**Figure 3:**
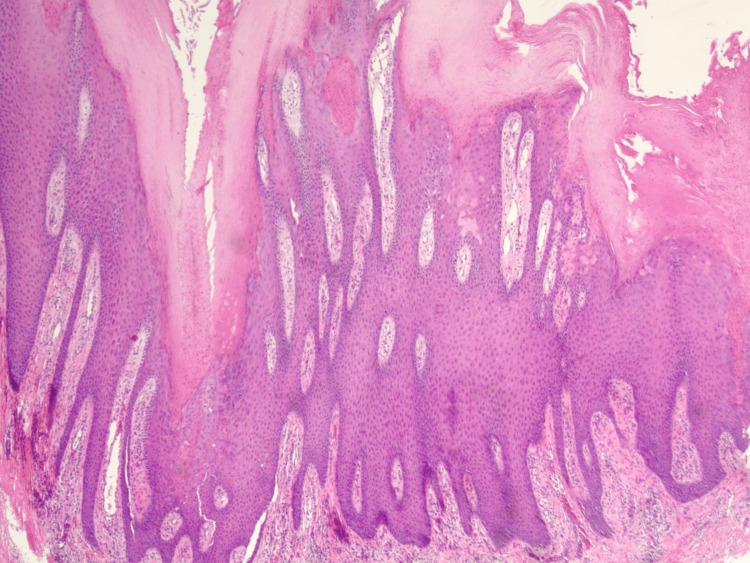
Multiple cornoid lamellae overlying sharply circumscribed zones of a diminished granular layer and scattered dyskeratotic keratinocytes. Rete ridges are elongated and evenly spaced. No significant cytologic atypia is present.

**Figure 4:**
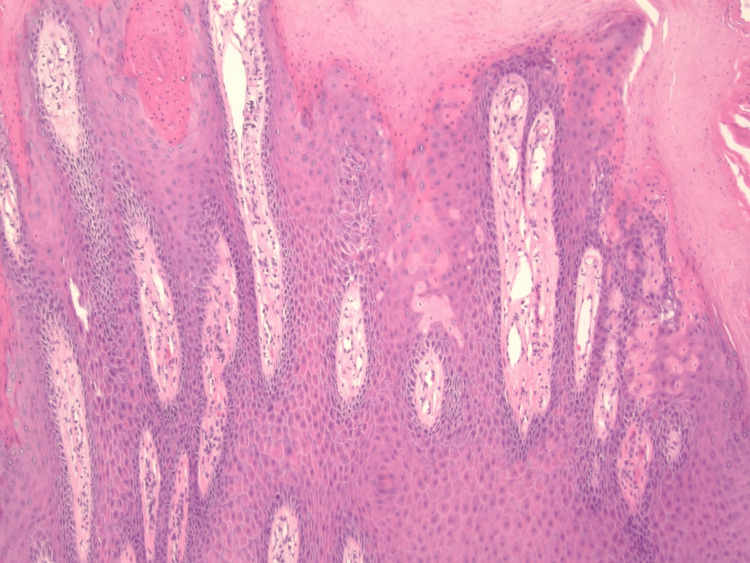
Close view of clustered cornoid lamellae with parakeratotic columns and subjacent hypogranulosis. The upper dermis shows a sparse lymphocytic infiltrate. No koilocytosis or invasion is identified.

Wide-field histology imaging was performed by tiling 20 overlapping fields (≈20-30% overlap) at 2× on a bright-field microscope with a digital camera. Tiles were stitched into a 5 × 4 mosaic using image-registration software (Hugin 2024.0.1), followed by global brightness/color normalization and rectangular cropping (Figure 2). Figure 3 presents the multiple cornoid lamellae overlying sharply circumscribed zones of a diminished granular layer and scattered dyskeratotic keratinocytes, and Figure 4 presents the close view of clustered cornoid lamellae with parakeratotic columns and subjacent hypogranulosis.

The patient tolerated the procedure well and had no immediate post-procedural complications, but was subsequently lost to follow-up.

## Discussion

PK comprises a spectrum of genodermatoses unified histologically by the cornoid lamella but varied in clinical forms (disseminated superficial actinic PK, DSAP; disseminated superficial PK, DSP; PK of Mibelli, PM; linear PK, LP; punctate PK/PK plantaris discreta, PP/PPD; PK palmaris et plantaris disseminata, PPPD; PK ptychotropica, PPt; genital PK, GP; giant PK; eruptive pruritic papular PK, EPPP; PK striata et reticulata, PSR; follicular PK; and zosteriform/segmental PK) [2].

A cornoid lamella is a column of parakeratotic stratum corneum overlying a focal zone of epidermal dysmaturation, typically with a thinned or absent granular layer and a subjacent zone of dyskeratosis. This distinctive stack of cornified cells is the histologic hallmark used to identify PK [3]. Importantly, cornoid lamellae are not entirely specific to PK; they represent a reaction pattern that signals localized abnormalities in keratinocyte maturation and can result from multiple upstream genetic or environmental causes. Similar parakeratotic columns may be seen in other conditions, including actinic keratosis, lichenoid keratosis, certain viral warts, callosities, and scars [4].

In recent years, germline mutations in mevalonate pathway enzymes have emerged as the major molecular drivers across variants of PK. Four genes in particular - *mevalonate kinase (MVK)*, *phosphomevalonate kinase (PMVK)*, *mevalonate diphosphate decarboxylase (MVD)*, and *farnesyl diphosphate synthase (FDPS)* - are recurrently found to be mutated, often in heterozygous form, in familial and sporadic PK. A landmark 2015 study by Zhang et al. sequenced 134 PK patients and found pathogenic variants in one of these four genes in 98% of familial cases and 73% of sporadic cases. This genetic convergence on the cholesterol/isoprenoid (mevalonate) pathway suggests a unifying biochemical theme in PK pathogenesis [5]. *MVK* mutations account for many classic DSAP cases, with some clinical presentations including fewer but larger plaques (>5 cm) [6]. Functionally, *MVK *appears to protect keratinocytes from stress-induced apoptosis; keratinocytes with *MVK *loss show abnormal calcium-induced differentiation and heightened UV-induced cell death [6], possibly explaining characteristic epidermal dysmaturation under each cornoid liamella. *MVD* and *PMVK *have been strongly linked to disseminated superficial PK, which presents as many small, relatively shallow lesions [7]. *PMVK *mutations are less common overall but show interesting phenotype correlations: for example, one study noted genital PK and even PRKT lesions arising in patients with *PMVK *variants [5]. *FDPS *mutations are the least frequent of the four, but tend to produce a very profuse DSAP-like eruption: patients with FDPS variants often have hundreds of tiny (<1 cm) annular lesions spread widely [5].

Recent description of localized PK due to bi-allelic inactivation of farnesyl‑diphosphate farnesyltransferase 1 (FDFT1, or squalene synthase) [8] is of particular interest due to the fact that *FDFT1 *lies just downstream of *FDPS *in the cholesterol biosynthesis pathway, reinforcing the theme of mevalonate-pathway insufficiency. *FDFT1*-deficient patients responded to topical cholesterol and statin (atorvastatin) therapy with marked clinical improvement [8], further validating the pathway's role and providing a pathogenesis-directed treatment clue.

PK displays a striking clonal mosaicism: even when a pathogenic variant is inherited, lesions typically arise only in certain skin clones, not universally. Genetic studies have shown that many PK lesions represent a “two-hit” phenomenon. A patient may carry a heterozygous germline mutation in, say, *MVK *or *PMVK*, but only develop lesions where a keratinocyte clone acquires a second hit (somatic mutation or loss of heterozygosity), knocking out the wild-type allele [9].

It is noteworthy that even purely “sporadic” PK cases often have a postzygotic mutation as the first hit. For example, some linear or localized cases have no mutation in blood but a heterozygous pathogenic variant restricted to lesional cells. In those, a second somatic event (within that clone) still seems requisite to produce the full lesion phenotype [10]. This aligns with the concept that normal skin is a genetic patchwork: studies of aged sun-exposed skin have revealed numerous microscopic mutant clones. Whole-mount analyses show that p53-mutant keratinocyte patches (60-3000 cells each) are present at >40 clones per cm², covering ~4% of an elderly epidermis [11]. Ultra-deep sequencing confirms that, by middle age, over a quarter of all skin cells carry UV- induced driver mutations in genes such as *Notch receptor 1 *(*NOTCH1*), *tumor protein p53 (TP53), FAT atypical cadherin 1 (FAT1)*, etc. [12] These findings raise the possibility that a PK lesion results when a mevalonate-pathway mutation occurs within an expanded pre-existing clone of keratinocytes. A large UV-selected clone harboring, say, a *NOTCH1 *mutation might provide a "fertile field" - if one cell in that field loses *MVK* or *MVD *function (second hit), the resultant PK plaque could propagate over the entire clone's area. This model remains hypothetical, but it fits the observed size variation of lesions and the frequent context of actinic (sun-exposed) distribution in DSAP. It also underscores that PK is fundamentally a clonal unit phenomenon - each lesion is an independent expansion of mutant cells [10].

Several hypotheses have been proposed to explain how a mutant clone of keratinocytes produces a cornoid lamella. One line of evidence points to altered terminal differentiation and keratinocyte apoptosis in the lesional epidermis with resulting hypogranulosis, aberrant expression of late differentiation markers (mislocalized transglutaminase-1 and expansion of basal keratin 14), and increased dyskeratosis aligning with the idea that a focal loss of mevalonate-pathway output could create an area of fragility where the epidermis cannot maintain its granular layer, leading to a stack of parakeratotic cells [9].

Another mechanistic layer involves autoinflammation. Recently, PK started to be viewed as an autoinflammatory keratinization disease (AiKD), meaning it combines an inherited barrier/differentiation defect with innate immune overactivation [13]. Considering known crosstalk between the mevalonate pathway and the inflammasome, demonstrated in some systemic autoinflammatory disorders, it is plausible that mevalonate-pathway haploinsufficiency may create a microenvironment of IL-1-mediated inflammation. Lesional skin often contains a lymphocytic infiltrate just beneath the cornoid lamella [3], and cases with coexisting inflammatory disorders lend support to an IL-1 link. In practice, IL-1 and related cytokines (IL-18, IL-36) have not been exhaustively studied in PK lesions, but the autoinflammatory classification and anecdotal therapeutic responses (some reports of isotretinoin or topical immunomodulators helping PK) keep this angle plausible [13].

Finally, the link to protein prenylation defects is intriguing. Mevalonate pathway enzymes ultimately produce farnesyl pyrophosphate and geranylgeranyl pyrophosphate, which prenylate many cellular signaling proteins (e.g., small GTPases). A local prenylation deficit in keratinocytes could disrupt key regulators of cell cycling, differentiation, and inflammasome control. The success of topical cholesterol and lovastatin (which provides substrate and reduces upstream metabolite accumulation) in treating PK supports the idea that restoring the metabolic balance can normalize keratinocyte behavior [10]. In one series, 2% lovastatin + 2% cholesterol ointment led to partial clearing of DSAP lesions [14], and in the new FDFT1 cases, a similar ointment caused significant improvement with no relapses [8]. This suggests that the cornoid lamella is, at least in those patients, reversible by correcting the prenylation and sterol metabolic deficit.

In summary, a cornoid lamella can be viewed as the skin's final common pathway for a certain kind of localized epidermal failure: keratinocytes (often in a clonal patch) are unable to terminally differentiate correctly, likely due to intrinsic genetic defects in isoprenoid metabolism, compounded by inflammatory signals. They undergo abnormal cornification and some apoptotic death, and the stratum corneum forms a columnar "stack" over the focus. This pattern can be triggered by different upstream causes (genetic or sometimes external), which is why we see cornoid lamellae outside of classical PK, albeit rarely [4].

PRKT was first defined in 2007 and manifests some architectural resemblance to a viral wart [1]. However, to date, only one PRKT case has definitively tested positive for HPV, leading to the conclusion that, while oncogenic HPV (like type 16) can occasionally be present in porokeratotic lesions (especially PRKT), it is likely not a primary driver in most cases of PK. Rather, we suggest that the metabolic/immunologic disturbance of PK might create a niche that is permissive for opportunistic HPV infection in some patients. Clinically, there is no routine role for antiviral therapy in PK, except perhaps in specific situations - for example, the imiquimod-responsive transplant case suggests one might consider an HPV search in unusually verrucous, progressive PK lesions under immunosuppression.

Most lesions described in the literature were solitary and arose on the extremities, although subsequent reports documented lesions on the buttocks, nipple, hand, and genitalia. Association with systemic conditions is uncommon; two patients had ankylosing spondylitis, and one had chronic lymphocytic leukemia.

Treatment has generally consisted of complete excision. Li et al. reported successful use of topical 5% fluorouracil for a lesion on the hand with no recurrence at 2.5 years [15], and Gaurav et al. treated a penile lesion with alternate‑day 5‑fluorouracil, resulting in complete resolution in six weeks [16]. Destructive modalities (cryotherapy, CO₂ laser) and systemic retinoids (acitretin) have been employed for multiple lesions or coexistence with PK. No cases of malignant transformation of PRKT have been documented, but known cases of squamous cell carcinoma arising in other forms of PK prompt caution and justify consideration of complete excision and long-term surveillance.

Our case report underscores the importance of considering PRKT in the differential diagnosis of solitary hyperkeratotic lesions and illustrates that even pedunculated lesions on the foot may represent this entity.

## Conclusions

PRKT is a rare epidermal acanthoma characterized histologically by confluent cornoid lamellae without the peripheral ridge or central atrophy typical of other PK variants. It should be considered in the differential diagnosis of solitary hyperkeratotic or verrucous lesions of the extremities, as accurate diagnosis relies on histopathologic evaluation. Management should be individualized based on clinical context and patient-specific factors, and clinical follow-up may be considered as appropriate.
